# Robot-Assisted Laparoscopic Versus Open Adenomyomectomy: Comparative Surgical and Reproductive Outcomes

**DOI:** 10.3390/jcm15062120

**Published:** 2026-03-10

**Authors:** Jung Hyun Park, Jae-Yen Song, Mee-Ran Kim, Youn-Jee Chung

**Affiliations:** Division of Reproductive Endocrinology, Department of Obstetrics and Gynecology, Soule St. Mary’s Hospital, College of Medicine, The Catholic University of Korea, Seoul 06591, Republic of Korea; jung0229@gmail.com (J.H.P.);

**Keywords:** adenomyosis, robotic surgical procedures, uterine diseases, fertility preservation, minimally invasive surgical procedures, adenomyomectomy

## Abstract

**Background/Objectives**: This study aimed to compare the surgical and reproductive outcomes between open and robot-assisted laparoscopic adenomyomectomy in women with symptomatic adenomyosis. **Methods**: This was a prospective cohort study conducted between 29 April 2017 and 21 December 2023 at a university-affiliated tertiary hospital in Seoul, Republic of Korea. The study included patients who underwent uterus-sparing adenomyomectomy for symptomatic adenomyosis. Eligible participants were premenopausal women seeking fertility preservation or symptomatic relief when medical therapy was ineffective and hysterectomy was not desired. Interventions consisted of open adenomyomectomy and robot-assisted laparoscopic adenomyomectomy. **Results**: A total of 76 patients were included: 21 underwent open adenomyomectomy (OA) and 55 received robot-assisted laparoscopic surgery (RLA). Patients in the OA group had larger adenomyotic lesions (7.1 ± 2.7 vs. 5.4 ± 1.7 cm, *p* = 0.001) and greater estimated blood loss (400 mL [200–700] vs. 300 mL [200–400], *p* = 0.05). Hospitalization was significantly shorter in the RLA group (β = −2.1 days, 95% CI −2.7 to −1.6, *p* < 0.001) after adjustment for lesion size. Overall delivery rates were comparable; however, the robotic cohort demonstrated fewer intraoperative endometrial exposures (10.0% vs. 55.6%, *p* = 0.033) and more favorable obstetric outcomes, with most deliveries reaching full term without postpartum complications. **Conclusions**: Compared with open surgery, robot-assisted laparoscopic adenomyomectomy was associated with reduced blood loss, shorter recovery, and favorable reproductive outcomes, supporting its value as a uterus-sparing treatment.

## 1. Introduction

Adenomyosis is characterized by ectopic infiltration of endometrial glands and stroma into the myometrium, resulting in myometrial hypertrophy, hyperplasia, and enlargement of the uterus [1]. Adenomyosis is associated with abnormal uterine bleeding, severe dysmenorrhea, and pelvic pain, but most importantly, it is a major cause of infertility [2,3]. Adenomyosis is predominantly diagnosed in premenopausal women between 35 and 50 years of age. However, with increasing maternal age and delayed childbearing, it has been increasingly recognized in women presenting with fertility concerns. In addition, the use of high-resolution ultrasonography and magnetic resonance imaging has improved diagnostic recognition, with a reported incidence of 4.12 per 1000 women in South Korea [4]. The risk factors of adenomyosis are early menarche, short menstrual cycles, elevated body mass index (BMI), increased parity, and prior uterine surgeries [5]. Although histologic examination following hysterectomy remains the only definitive method for confirming adenomyosis, clinicians attempt to preserve the uterus in several ways, including uterine artery embolization, adenomyomectomy, and other medical treatments [6].

Alternative treatment options should be considered prior to hysterectomy in women who require fertility preservation. Symptomatic relief can be achieved with pharmacological therapy, including nonsteroidal anti-inflammatory drugs (NSAIDs), gonadotropin-releasing hormone agonists (GnRHa), progestins, and oral contraceptives (OCs) [7,8]. Nevertheless, if medical management proves ineffective and the patient intends to conceive in the future, excisional surgery should be considered as a feasible treatment option. Adenomyomectomy remains a challenging area of surgical research, and a standardized approach for conservative treatment of adenomyosis has yet to be established [9]. Although difficult, complete removal of adenomyotic lesions in cases of focal adenomyosis is worth attempting, but not in cases of diffuse adenomyosis. The extent of incomplete or excessive excision should be determined by the surgeon’s judgment [10]. Although conventional methods rely on open adenomyomectomy, recent efforts have explored laparoscopic approaches aligned with the principles of minimally invasive surgery. However, studies have demonstrated that laparoscopic adenomyomectomy is associated with a high risk of uterine rupture during pregnancy [11].

Robotic surgery is regarded as the most advanced form of minimally invasive surgery and is widely used in gynecology to overcome difficulties encountered in conventional laparoscopic technology [12]. A robotic system offers a three-dimensional perspective and allows for seven degrees of hand movement, enabling surgeons to achieve high accuracy and a performance comparable to that of traditional open procedures. In addition, the use of intraoperative real-time ultrasonography complements the tactile sensation, which cannot be achieved in robotic surgeries. Especially in the novel technique of adenomyomectomy, checking the depth of resected adenomyotic lesions with real-time ultrasonography assists in minimizing the opening of the endometrium [10,13].

Studies examining the surgical feasibility of robotic adenomyomectomy are scarce. Furthermore, no studies have compared the surgical outcomes of the open and robotic adenomyomectomy procedures. This study aimed to evaluate and compare the surgical and obstetric outcomes of robot-assisted laparoscopic adenomyomectomy with those of patients who underwent adenomyomectomy using a conventional open approach.

## 2. Materials and Methods

### 2.1. Patients

This study included patients who underwent adenomyomectomy from 29 April 2017 to 21 December 2023 at the fibroid center in a university-affiliated tertiary hospital in the Republic of Korea. Patients underwent either open or robot-assisted laparoscopic adenomyomectomy. Candidates were selected for surgery based on the following criteria: (1) women planning for pregnancy and requiring fertility preservation, (2) women experiencing symptoms such as heavy vaginal bleeding or pelvic pain who did not respond to pharmacological interventions and were averse to hysterectomy, and (3) premenopausal women younger than 45 years of age.

The diagnosis of adenomyosis was initially suspected on transvaginal ultrasonography based on features consistent with the Morphological Uterus Sonographic Assessment consensus criteria [14,15]. Subsequently, all cases were confirmed by pelvic magnetic resonance imaging (MRI), and only patients demonstrating established imaging findings of adenomyosis—such as junctional zone thickening (≥12 mm), irregular or ill-defined junctional zone, and/or high-signal intensity myometrial foci—were included for surgical decision-making [16].

Exclusion criteria included patients who underwent laparoscopic surgery and those who underwent concurrent adenomyomectomy and myomectomy in order to ensure a more precise comparison of postoperative changes in uterine wall thickness, adenomyotic lesion size, and clinical outcomes.

In the open adenomyomectomy (OA) group (*n* = 21), 11 patients underwent surgery for fertility preservation, whereas 10 had symptomatic adenomyosis and elected uterine preservation rather than hysterectomy. In the robot-assisted laparoscopic surgery (RLA) group (*n* = 55), 25 patients underwent surgery for fertility preservation, and 30 had symptomatic adenomyosis but declined hysterectomy. Uterine artery embolization was not routinely offered prior to adenomyomectomy in either group.

This study was approved by the Institutional Review Board of the Catholic University of Korea (IRB Number: KC17OESI0238) and was performed according to the principles set out in the Declaration of Helsinki. Participants were enrolled in a prospective cohort study with informed consent, without random allocation, as no intervention was administered.

### 2.2. Study Parameters and Assay Methods

Patient age, parity, previous operative history, BMI, and pelvic pain-related visual analog scale (VAS) scores were investigated. To determine the exact location and size of the adenomyotic lesion, all patients underwent enhanced pelvic MRI preoperatively. Serum cancer antigen 125 (CA 125) level was evaluated before and after the surgery.

Hemoglobin drop was evaluated by a CBC follow-up on postoperative day 1. Jackson-Pratt drain placement was optional, and patients without complications were discharged after the drainage removal. The first outpatient visit, scheduled 2 weeks after surgery, was conducted for wound care and pathological evaluation. At the second outpatient visit, 3 months after surgery, pelvic ultrasonography was performed, serum CA 125 levels were reassessed, and improvement in pelvic pain was evaluated using the VAS. Based on these comprehensive findings, patients were counseled that attempts at conception could be initiated from 6 to 12 months postoperatively.

A comprehensive chart review was conducted in 2024, corresponding to 4–6 years after the index surgery, to evaluate postoperative medical treatments, pregnancy status, and delivery outcomes. For patients followed at other institutions, additional information regarding pregnancy status, obstetric outcomes, delivery mode, and complications, including uterine rupture, was collected through telephone interviews.

### 2.3. Open Adenomyomectomy

Surgery was performed under general anesthesia in a dorsal supine position. A sterile 16-French pediatric Foley catheter was inserted into the endometrial cavity to monitor potential endometrial injury using the indigo carmine test. A 13–15 cm Pfannenstiel incision was made at a height of level 1 finger above the pubis, and the adenomyoma was localized by visual inspection with intraoperative ultrasonography and preoperative MRI review. Diluted vasopressin (10 IU/100 mL saline) was injected into the serosa and myometrium. A horizontal or vertical ‘cold-cut’ incision was made over the adenomyoma depending on its size and extent, while preserving 1–1.5 cm of overlying myometrium. Following excision, the innermost myometrial layers were approximated with 2-0 STRATAFIX™ barbed sutures (Ethicon Inc., Somerville, NJ, USA), and the remaining myometrium was repaired in interrupted layers using 2-0 Vicryl™ polyglactin 910 sutures (Ethicon Inc., Somerville, NJ, USA) to avoid laceration and dead space. The serosal layer was then closed with a baseball suture, and all specimens were submitted for histopathological confirmation.

### 2.4. Robot-Assisted Laparoscopic Adenomyomectomy

**Figure 1:**
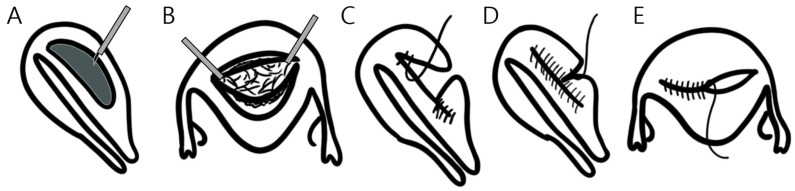
Surgical procedures of robot-assisted laparoscopic adenomyomectomy: a novel method. (**A**) Diluted vasopressin was injected using the laparoscopic aspiration needle between the serosa and myometrium surrounding the adenomyoma. (**B**) The adenomyotic lesion was excised using a ‘cold-cut’ technique with either curved monopolar scissors or a monopolar spatula, assisted by a tenaculum forceps holding the tissue. (**C**) The defect between the anterior and posterior innermost myometrial layers was closed using a 2-0 Stratafix suture. (**D**) After the anteroposterior planes were sutured, the two sides were approximated using a Polydioxanone 2-0 suture. (**E**) Serosal closure was performed in the baseball technique.

Surgery was performed under general anesthesia in the dorsal lithotomy position using Da Vinci^®^ Surgical system (Si or Xi platform; Intuitive Surgical Inc., Sunnyvale, CA, USA). One 12 mm trocar was inserted using the open Hasson method, followed by two 8 mm trocars in the bilateral lower quadrants and a 5 mm trocar in the right upper quadrant for the assistant. Preferred instruments included curved monopolar scissors, Maryland bipolar forceps, a cautery spatula, tenaculum forceps, and mega-needle drivers. After identifying the adenomyotic lesion, diluted vasopressin was injected between the serosa and myometrium (Figure 1A). Using curved monopolar scissors without electrocauterization (also called a ‘cold-cut’ technique), we performed a horizontal or vertical incision over the vertex of the adenomyoma. The adenomyotic tissue was grasped using tenaculum forceps to provide traction, and dissection was continued using monopolar curved scissors or spatula (Figure 1B).

Intraoperative real-time pelvic ultrasonography was employed to maintain a residual myometrial thickness of 1.0–1.5 cm. The myometrial defect was closed with 2-0 STRATAFIX™ barbed sutures (Ethicon Inc., Somerville, NJ, USA) to approximate the anterior and posterior layers without leaving dead space (Figure 1C), followed by serosal closure using a continuous 2-0 polydioxanone (PDS) suture in a baseball fashion (Figure 1E). Excised specimens were retrieved by endoscopic bag morcellation and submitted for histopathological confirmation.

### 2.5. Statistical Analysis

Continuous variables were summarized as mean ± standard deviation or median with interquartile range, and categorical variables were presented as counts and percentages. Group comparisons were performed using the independent *t*-test or Mann–Whitney U test for continuous variables, and the χ^2^ or Fisher’s exact test for categorical variables. Perioperative outcomes were analyzed with linear regression models adjusted for preoperative adenomyosis lesion size. The crude and adjusted odds ratios (ORs) for reproductive outcomes after adenomyomectomy were determined using logistic regression analysis and presented as point estimates with 95% confidence interval (CI). Multivariable models were adjusted for lesion size and endometrial exposure. To strengthen causal inference, inverse probability weighting with covariate balancing propensity scores (IPW-CBPS) combined with regression adjustment (RA) was applied [17,18], and covariate balance was assessed using standardized mean differences and variance ratios. All analyses were two-sided, with *p* < 0.05 considered statistically significant. Statistical analyses were conducted using Stata 17.0 (College Station, TX, USA), while IPW-CBPS with RA was performed using KMATCH, a community-contributed Stata command [17].

### 2.6. Study Endpoint

The primary endpoint was to assess surgical performance between open and robotic adenomyomectomy, primarily in terms of intraoperative blood loss. Secondary analyses examined postoperative pregnancy outcomes.

## 3. Results

### 3.1. Patient Characteristics

A total of 76 patients were included: 21 underwent OA and 55 received RLA. Baseline age and BMI did not differ significantly between groups (Table 1). Patients in the OA group had larger adenomyotic lesions (7.1 ± 2.7 vs. 5.4 ± 1.7 cm, *p* = 0.001) and higher baseline CA 125 levels (302.8 ± 493.5 vs. 104.8 ± 112.6 U/mL, *p* = 0.004). The proportion of married patients was higher in the open cohort (61.9% vs. 29.1%, *p* = 0.008), whereas nulligravida status and abortion history did not differ significantly.

### 3.2. Surgical Performances and Postoperative Clinical Outcomes

Perioperative outcomes are summarized in Table 2. Median estimated blood loss (EBL) was greater in the OA group (400 mL [200–700] vs. 300 mL [200–400], *p* = 0.05). Mean operation time was significantly longer in the RLA group (283.8 ± 77.0 vs. 240.2 ± 61.3 min, *p* = 0.02). Blood transfusion was required more frequently after open surgery (42.9% vs. 14.6%, *p* = 0.01), and intraoperative endometrial exposure was more common (47.6% vs. 23.6%, *p* = 0.04). Hospital stay was significantly shorter in the robotic cohort (median 3 days [2–3] vs. 5 days [5–6], *p* < 0.001). Postoperative CA 125 reduction and pain improvement assessed by VAS did not differ significantly between groups.

Based on linear regression analysis adjusted for preoperative lesion size, the robotic approach was associated with a nonsignificant trend toward lower blood loss (β = −199.4 mL, 95% CI −418.4 to 19.6, *p* = 0.07) and shorter hospitalization (β = −2.1 days, 95% CI −2.7 to −1.6, *p* < 0.001) (Table 2). Adjusted analysis for transfusion and endometrial exposure percentage did not reveal significant differences.

Surgical findings, including revised American Society for Reproductive Medicine (rASRM) scores (Supplementary Table S1), revealed that adenomyotic lesions were predominantly located posteriorly in both groups (80.95% in the OA group vs. 72.73% in the RLA group, *p* = 0.459). Among patients with posterior wall adenomyosis, coexistence with endometriosis was frequent in both groups (100% in the OA group vs. 78.1% in the RLA group, *p* = 0.105). The mean rASRM score did not differ significantly (41.1 ± 46.9 vs. 40.3 ± 51.0, *p* = 0.949).

### 3.3. Postoperative Reproductive Outcomes

#### 3.3.1. Postoperative Management and Reproductive Attempts

In the OA group (n = 21), six patients received a levonorgestrel-releasing intrauterine system (LNG-IUS), two were treated with Dienogest or continuous oral contraceptives (OCs), one was treated with GnRHa, nine attempted pregnancy without further therapy, and one later underwent hysterectomy (Supplementary Figure S1). In the RLA group (n = 55), 29 patients were treated with LNG-IUS, nine received Dienogest or continuous OCs, and ten attempted conception directly. Additionally, one patient underwent uterine artery embolization (UAE), and another required an open myomectomy within four years of the index surgery.

#### 3.3.2. Postoperative Obstetric Outcomes

Postoperative reproductive outcomes are presented in Table 3. Conception attempts were more frequent in the OA group (42.9% vs. 18.2%, *p* = 0.026). All patients were counseled to attempt conception at least 6 months after surgery, and in both groups, the interval between surgery and conception ranged from 1 to 3 years. Among these patients, intraoperative endometrial exposure occurred more often in the open cohort (55.6% vs. 10.0%, *p* = 0.033). Delivery rates were comparable between the two groups (44.4% vs. 60.0%, *p* = 0.498).

All deliveries in both groups were performed via cesarean section. In the OA group, all four deliveries resulted in preterm births (<37 weeks), including one twin pregnancy complicated by threatened preterm labor (TPL), two singleton pregnancies with a history of incompetent internal os of the cervix (IIOC) followed by TPL, and one preterm cesarean delivery due to severe preeclampsia. In contrast, in the RLA group, four of six deliveries were full-term. One preterm cesarean section was performed due to preeclampsia, and one patient with a history of IIOC experienced singleton TPL.

No cases of uterine rupture or placenta accreta spectrum disorders were observed in either cohort. Rescue cerclage was required in two open cases and one robotic case. Postpartum complications occurred in two open patients (one wound dehiscence and one mild postpartum hemorrhage), while no complications were reported in the RLA group.

Logistic regression analysis was conducted to evaluate the association between surgical modality and subsequent pregnancy (Table 4). In univariable analysis, the odds ratio (OR) for pregnancy after robotic surgery compared with open surgery was 0.88 (95% CI 0.14–5.58, *p* = 0.89). After adjustment for preoperative adenomyotic lesion size and endometrial exposure, the association remained nonsignificant (adjusted OR = 0.48, 95% CI 0.05–4.35, *p* = 0.52). In the doubly robust model using IPW-CBPS and RA, no significant difference was observed (adjusted OR = 0.81, 95% CI 0.57–1.15, *p* = 0.23).

To assess the adequacy of confounding control, baseline covariates were compared before and after weighting with IPW-CBPS (Table 5). Standardized mean differences and variance ratios for lesion size and endometrial exposure improved markedly after weighting, and cumulative probability plots confirmed substantial covariate balance (Supplementary Figure S2).

## 4. Discussion

This study reviewed 76 cases of open and robot-assisted laparoscopic adenomyomectomy performed over a 7-year period at a tertiary university hospital in Korea. Compared with the open approach, the robotic procedure was associated with significantly lower EBL, shorter hospital stays, and less frequent intraoperative endometrial exposure. Because baseline adenomyotic lesions were larger in the OA group, linear regression was performed to adjust for lesion size, and hospitalization remained significantly shorter in the RLA group after adjustment. In terms of postoperative management, most patients in both groups were treated with LNG-IUS, while conception attempts were more common in the OA group. Although postoperative reproductive outcomes did not differ significantly between groups, the robotic cohort showed favorable trends, with most deliveries reaching full term and no postpartum complications.

From a clinical perspective, these findings should be interpreted within the context of surgical trade-offs and baseline disease severity. Although RLA was associated with longer operative times, this likely reflects the technical complexity of the robotic platform and may represent an acceptable trade-off for reduced intraoperative blood loss and shorter hospitalization compared with the OA group.

The lower incidence of intraoperative endometrial exposure in the RLA group may partially be attributed to the magnified three-dimensional visualization system and enhanced instrument articulation inherent to robotic surgery. These technical advantages facilitate precise lesion–myometrium discrimination and meticulous uterine reconstruction, particularly in anatomically challenging regions such as the posterior uterine wall. Consequently, preservation of endometrial integrity and optimization of myometrial repair may have been more consistently achieved in the RLA group. In contrast, the larger baseline lesion size observed in the OA group likely necessitated more extensive excision, which may have increased the likelihood of endometrial breach and greater myometrial tissue removal. Notably, however, adjusted analyses accounting for lesion size did not demonstrate a statistically significant difference in endometrial exposure rates between groups. This suggests that baseline disease severity may have partially contributed to the observed crude differences. In cases of intraoperative endometrial exposure, hysteroscopic evaluation of the endometrial cavity was routinely performed prior to completion of the procedure to assess cavity integrity and reduce the risk of postoperative intrauterine adhesion formation, particularly in patients desiring future fertility.

In addition, most patients in both groups received postoperative hormonal suppression, typically consisting of short-term GnRHa therapy followed by delayed insertion of an LNG-IUS. In our cohort, LNG-IUS insertion was planned at least 6 months postoperatively following initial GnRHa injection, balancing recurrence prevention with uterine healing considerations. Short-term administration of GnRHa suppresses residual adenomyotic activity and induces a hypoestrogenic state that may promote myometrial remodeling during the early healing phase [19,20]. Subsequent maintenance therapy with LNG-IUS provides sustained local progestogenic effects, alleviating abnormal uterine bleeding and dysmenorrhea while potentially limiting disease progression and recurrence [21].

This pioneering study compared open adenomyomectomy with robotic surgery, which represents the most advanced form of minimally invasive surgical technology, thereby addressing limitations in the prior literature. Notably, our findings differ from those of Shim et al., who compared laparoscopic and robotic adenomyomectomy and reported trends toward reduced blood loss and longer operative and hospital stay times in the robotic group, although without statistical significance [22]. Their study excluded open adenomyomectomy cases, analyzing only 21 laparoscopic and 22 robotic procedures, and included patients undergoing concomitant myomectomy, potentially introducing heterogeneity in surgical outcomes. In contrast, our study incorporated open cases and enhanced the accuracy of comparisons by excluding patients who underwent simultaneous myomectomy and adenomyomectomy, while also adjusting for baseline lesion size to reduce confounding. Zhu et al. compared laparotomic and laparoscopic adenomyomectomy and reported higher long-term efficacy and lower recurrence in the open cohort [23]. Whereas their analysis primarily focused on symptom control and recurrence, our study further examined intraoperative structural preservation, including endometrial exposure, and its potential implications for subsequent reproductive outcomes. Furthermore, surgical precision in our cohort was enhanced by real-time intraoperative ultrasonography, which may have contributed to differences in intraoperative findings. Collectively, these methodological distinctions may explain variations across studies and highlight the need for further comparative investigations incorporating standardized reproductive end-points.

Our findings of favorable reproductive outcomes in the robotic cohort may be explained by the significantly lower rate of intraoperative endometrial exposure compared with the OA group. The predominance of full-term deliveries without postpartum complications in the robotic group suggests improved uterine healing and structural integrity with this approach. Although logistic regression indicated slightly lower odds of pregnancy in the robotic group, this association was not statistically significant and was likely attributable to the smaller number of patients attempting conception. Importantly, no uterine rupture was observed in either group, despite prior reports identifying rupture during pregnancy as the most serious concern after adenomyomectomy [24,25,26]. This reassuring finding may reflect the safety of our novel technique, which incorporates real-time intraoperative ultrasonography and preserves a residual myometrial thickness of 1.0–1.5 cm from the endometrium [13,27,28]. In robotic surgery, intraoperative ultrasonography and high-definition 3D visualization enhance lesion–myometrium discrimination, thereby minimizing endometrial injury and optimizing uterine reconstruction [29,30]. Collectively, these results underscore that surgical precision and preservation of the endometrium, rather than surgical modality alone, are critical determinants of reproductive safety in uterus-preserving surgery.

This study offers several notable strengths in addition to its novelty. First, this study improved the accuracy of surgical outcome comparisons by excluding patients who underwent concomitant myomectomy, thereby including only those who received adenomyomectomy. Second, baseline differences in adenomyotic lesion size were addressed through adjusted analyses, allowing more accurate interpretation of perioperative and intraoperative outcomes. Third, to our knowledge, this is the first study to incorporate intraoperative endometrial exposure as an analytic variable in adenomyomectomy and to examine its association with subsequent obstetric outcomes. Furthermore, surgical safety was reinforced through the routine use of intraoperative ultrasonography and hysteroscopic evaluation in cases of endometrial exposure, as well as through a standardized postoperative hormonal protocol consisting of short-term GnRHa followed by delayed LNG-IUS insertion. Together with the relatively long follow-up period for reproductive outcomes, these methodological features strengthen the internal validity of our findings.

Several limitations should be acknowledged. Although our institution is the nation’s first dedicated fibroid center and has accumulated a substantial number of adenomyomectomy cases, many patients underwent surgery for symptomatic relief rather than fertility preservation, resulting in a limited number of conception attempts and deliveries. This reduced the statistical power and precision of reproductive analyses, as reflected by the wide confidence intervals in the regression models. Accordingly, the finding that all deliveries in the OA group were preterm should be interpreted cautiously, particularly given the small sample size and larger baseline lesion size in that group. Despite the use of Fisher’s exact test for small cell counts, the limited number of events remains an inherent constraint.

Second, although the exclusion of patients who underwent concomitant myomectomy enhanced internal validity by enabling a more focused comparison of adenomyomectomy outcomes, it may limit generalizability to broader clinical settings in which adenomyosis and leiomyomas frequently coexist. In addition, the retrospective design precluded systematic documentation of the mode of conception, which may constrain interpretation of reproductive outcomes. Despite these limitations, our study demonstrates that robotic adenomyomectomy, compared with open surgery, resulted in lower blood loss and favorable reproductive outcomes, supporting its role as a feasible uterus-sparing treatment option.

## 5. Conclusions

Compared with open surgery, robot-assisted laparoscopic adenomyomectomy resulted in less blood loss, shorter recovery, and improved reproductive outcomes, supporting its role in fertility preservation.

## Figures and Tables

**Table 1:** Baseline characteristics of open and robot-assisted laparoscopic adenomyomectomy patients.

	OA (*n 21*)	RLA (*n 55*)	*p*-Value
Age (yr)	37.8 ± 2.6	36.1 ± 4.9	0.151
BMI (kg/m^2^)	21.8 ± 3.9	22.5 ± 3.7	0.479
Adenomyosis lesion size (cm)	7.1 ± 2.7	5.4 ± 1.7	0.001 *
CA 125 (U/mL)	302.8 ± 493.5	104.8 ± 112.6	0.004 *
Married patient (%)	61.9 (13/21)	29.1 (16/55)	0.008 *
Nulligravida (%)	80.9 (17/21)	72.7 (40/55)	0.459
Abortion history (%)	14.3 (3/21)	23.6 (13/55)	0.371

An asterisk indicates statistical significance. Values are presented as mean ± standard deviation or percentage (number of patients). Abbreviations: BMI, body mass index; CA 125, Cancer Antigen 125.

**Table 2:** Surgical performances and postoperative clinical outcomes of open and robot-assisted laparoscopic adenomyomectomy.

	OA (*n 21*)	RLA (*n 55*)	*p*-Value	Adenomyosis Lesion Size-Adjusted		
				OA (*n 21*)	RLA (*n 55*)	*p*-Value
**Primary Endpoint**						
EBL (mL)	400 (200–700)	300 (200–400)	0.05	Ref	−199.4 (−418.4, 19.6)	0.07
**Secondary Endpoint**						
Operation Time (min)	240.2 ± 61.3	283.8 ± 77.0	0.02	Ref	57.1 (17.3, 97.0)	0.01
Hemoglobin Drop	2.0 ± 1.2	2.1 ± 1.3	0.62	Ref	−0.2 (−0.8, 0.5)	0.62
Blood Transfusion (%)	9 (42.9)	8 (14.6)	0.01	Ref	0.37 (0.11, 1.27)	0.12
Endometrium Exposure Cases (%)	10 (47.6)	13 (23.6)	0.04	Ref	0.54 (0.17, 1.70)	0.29
Length of Hospital Days ^#^	5 (5–6)	3 (2–3)	<0.001	Ref	−2.1 (−2.7, −1.6)	<0.001
CA 125 Drop (U/mL) *	66.2 (1.9–169.7)	51.3 (21.3–148.2)	0.12	Ref	18.1 (−1.0, 37.2)	0.06
Postoperative Pain VAS Score Drop **	9 (8.5–10)	8 (7–10)	0.19	Ref	0.0 (−0.2, 0.2)	0.93

Values are presented as mean ± standard deviation or number (%). Abbreviations: EBL, estimated blood loss; CA 125, Cancer Antigen 125; VAS, visual analog scale. Linear regression, adjusted for the preoperative size of the adenomyosis lesion. ^#^ Poisson regression. * Additionally adjusted for pre-operative CA 125. ** Poisson regression, additionally adjusted for pre-operative VAS.

**Table 3:** Postoperative Obstetric Outcomes of Open and Robot-Assisted Laparoscopic Adenomyomectomy.

	OA		RLA		*p* Value
	n	%	n	%	
Attempt to conceive	9	42.86 (9/21)	10	18.18 (10/55)	0.026 *
Endometrium exposure	5	55.55 (5/9)	1	10.0 (1/10)	0.032 *
Abortion	0	0.00	0	0.00	
Delivery	4	44.44 (4/9)	6	60.0 (6/10)	0.497
Preterm delivery (<36 + 6 weeks)	4	100.0 (4/4)	2	33.33 (2/6)	0.08 ^‡^
Full-term delivery (>37 + 0 weeks)	0	0.0	4	66.66 (4/6)	
Rescue cerclage	2		1		
Complication	2 †		0		
Uterine rupture	0	0.00	0	0.00	

An asterisk indicates statistical significance. † Complication: 1 case of wound dehiscence and 1 case of mild postpartum bleeding resolved via conservative treatment. ^‡^ *p* value by Fisher’s Exact test.

**Table 4:** Odds ratio for pregnancy after adenomyomectomy according to the type of operation.

	Univariable Logistic Regression			Multivariable Logistic Regression			IPW-CBPS and RA		
	OR	95% CI	*p* Value	Adjusted OR	95% CI	*p* Value	Adjusted OR	95% CI	*p* Value
OA	Ref			Ref			Ref		
RLA	0.88	0.14–5.58	0.89	0.48	0.05–4.35	0.52	0.81	0.57–1.15	0.23

Abbreviations: OA, open adenomyomectomy; RLA, robot-assisted laparoscopic surgery; IPW-CBPS, inverse probability weighting with weights estimated from the covariate balance propensity score; RA, regression adjustment; OR, odds ratio; CI, confidence interval. Multivariable logistic regression adjusted for the preoperative size of the adenomyosis lesion and endometrial exposure. The covariate balancing propensity score (CBPS) was based on the preoperative size of the adenomyosis lesion and endometrial exposure. Regression adjustment (RA) for the preoperative size of the adenomyosis lesion and endometrial exposure.

**Table 5:** Summary of the balance of variables before and after IPW-CBPS.

	Standardized Mean Difference		Variance Ratio	
	Before IPW-CBPS	After IPW-CBPS	Before IPW-CBPS	After IPW-CBPS
The preoperative size of the adenomyosis lesion	−0.54	−0.06	1.11	1.19
Endometrial exposure	−0.56	−0.02	0.59	0.94

Abbreviations: IPW-CBPS, inverse probability weighting with weights estimated from the covariate balance propensity score.

## Data Availability

The original contributions presented in the study are included in the article/Supplemental Material; further inquiries can be directed at the corresponding author.

## Supplementary Materials

The following supporting information can be downloaded at https://www.mdpi.com/article/10.3390/jcm15062120/s1. Figure S1: Postoperative management and reproductive attempts of open and robotic surgery groups; Figure S2: Cumulative density function of probability of treatment assignment; Table S1: Baseline Characteristics of Open and Robot-assisted laparoscopic adenomyomectomy patients based on the Surgical Findings.

## Abbreviations

The following abbreviations are used in this manuscript:

BMI	Body Mass Index
NSAIDs	Nonsteroidal Anti-Inflammatory Drugs
GnRHa	Gonadotropin-Releasing Hormone agonists
OCs	Oral Contraceptives
MRI	Magnetic Resonance Imaging
VAS	Visual Analog Scale
CBC	Complete Blood Count
CA 125	Cancer Antigen 125
EBL	Estimated Blood Loss
PDS	Polydioxanone
LNG-IUS	Levonorgestrel-Releasing Intrauterine System
UAE	Uterine Artery Embolization
ASRM	American Society for Reproductive Medicine
IPTW-CBPS	Inverse Probability Weighting with Weights Estimated from the Covariate Balance Propensity Score
RA	Regression Adjustment
TPL	Threatened Preterm Labor
IIOC	Incompetent Internal os of the Cervix
OR	Odds Ratio
