# Supplementary material for: Robot-Assisted Laparoscopic Versus Open Adenomyomectomy: Comparative Surgical and Reproductive Outcomes
Source: J Clin Med. 2026 Mar 10;15(6):2120. doi: 10.3390/jcm15062120 (PMC13027369; doi:10.3390/jcm15062120)
Supplement: Supplementary file 1 [file jcm-15-02120-s001.zip › jcm-4159619-supplementary.pdf]

Supplemental materials

Robot-Assisted Laparoscopic versus Open Adenomyomectomy: Comparative Surgical and Reproductive Outcomes

Jung Hyun Park, Jae-Yen Song, Mee-Ran Kim, Youn-Jee Chung*

Department of Obstetrics and Gynecology, College of Medicine, The Catholic University of Korea, Seoul, Republic of Korea

Corresponding Author: Youn-Jee Chung MD,PhD

Division of Reproductive Endocrinology, Department of Obstetrics and Gynecology, College of Medicine, The Catholic University of Korea, 222, Banpo-daero, Seocho-gu, Seoul, 06591, Republic of Korea

Phone: +82-10-8964-7232, E-mail: porshe80@catholic.ac.kr

A Table of Contents

Supplementary Figures

Supplementary Figure S1. Postoperative management and reproductive attempts of open and robotic surgery groups

Supplementary Figure S2. Cumulative density function of probability of treatment assignment

Supplementary Table

Supplementary Table S1. Baseline Characteristics of Open and Robot-assisted laparoscopic adenomyomectomy patients based on the Surgical Findings

Supplementary Figure S1. Postoperative management and reproductive attempts of open and robotic surgery groups

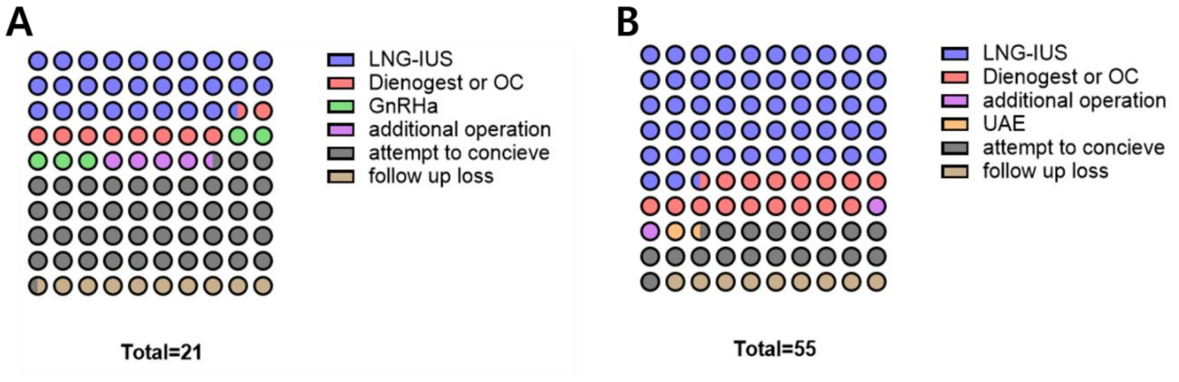

(A) Among 21 OA group patients, six were medically treated via LNG-IUS, two were taking Dienogest or continuous Ocs, one was treated by GnRHa, nine were trying pregnancy, and one patient underwent open hysterectomy three years after the initial surgery. (B) In the RLA group, 29 out of 55 patients were managed by LNG-IUS, nine were treated by Dienogest or continuous OCs, ten were attempted to conceive, one underwent UAE, and one patient underwent additional open myomectomy in four years.

Abbreviations: LNG-IUS, levonorgestrel-releasing intrauterine system; OCs, Oral Contraceptives; GnRHa, gonadotropin-releasing hormone agonist; UAE, uterine artery embolization.

Supplementary Figure S2. Cumulative density function of probability of treatment assignment

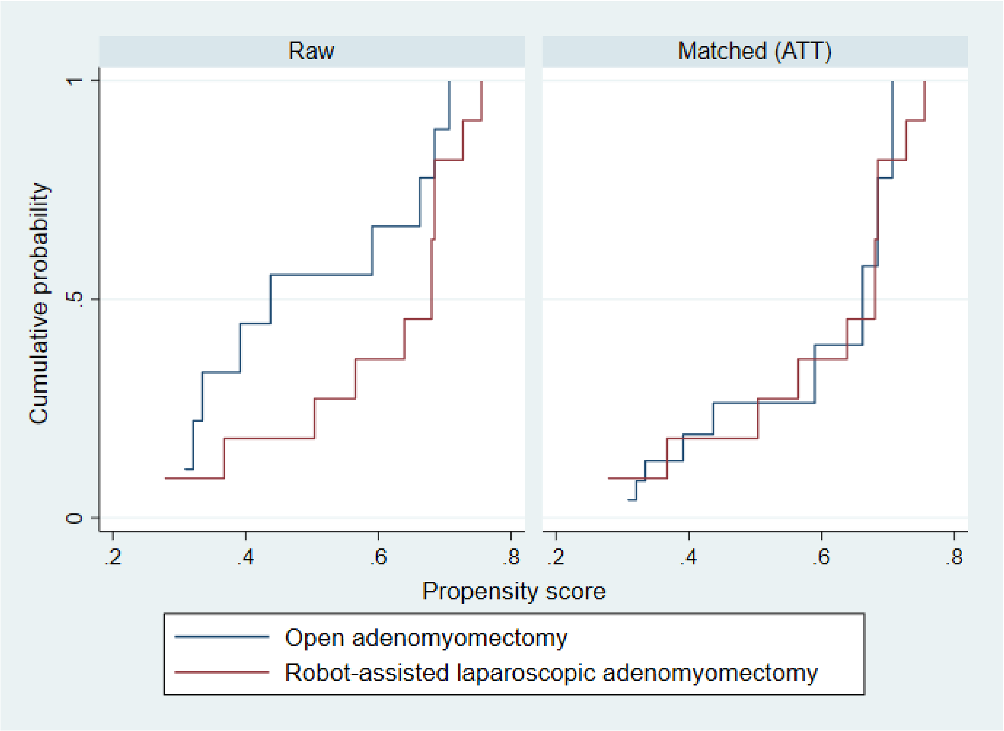

Abbreviations: ATT, average treatment effect on the treated

Supplementary Table S1. Baseline Characteristics of Open and Robot-assisted laparoscopic adenomyomectomy patients based on the Surgical Findings

	**OA** (***n 21***)	**RLA** (***n 55***)	**P-value**
Adenomyosis lesion on anterior uterus wall (%)	19.05 (4/21)	27.27 (15/55)	0.459
Adenomyosis lesion on posterior uterus wall (%)	80.95 (17/21)	72.73 (40/55)	0.459
Endometriosis combined cases (%)	47.6 (10/21)	58.18 (32/55)	0.407
Em combined cases with adenomyosis lesion on posterior uterus wall (%)	100.0 (10/10)	78.12 (25/32)	0.105
Endometriosis rASRM score	41.14±46.93	40.33±50.95	0.949

Values are presented as mean ± standard deviation or percentage (number of patients).

Abbreviations: rASRM, revised American Society for Reproductive Medicine.
